# Survey of Zoonotic Bacterial Pathogens in Native Foxes in Central Chile: First Record of *Brucella canis* Exposure

**DOI:** 10.3390/ani11071980

**Published:** 2021-07-02

**Authors:** Nicolás Galarce, Sebastián de la Fuente, Beatriz Escobar, Phillip Dettleff, Pedro Abalos, Juan Carlos Hormazábal, Roberto Flores, Nicole Sallaberry-Pincheira, Víctor Martínez

**Affiliations:** 1 Departamento de Medicina Preventiva Animal, Facultad de Ciencias Veterinarias y Pecuarias, Universidad de Chile, Santiago 8820808, Chile; ngalarce@ug.uchile.cl (N.G.); seba.delafuenteg@gmail.com (S.d.l.F.); beatrizescob@gmail.com (B.E.); pabalos@uchile.cl (P.A.); 2 Departamento de Fomento de la Producción Animal, Facultad de Ciencias Veterinarias y Pecuarias, Universidad de Chile, Santiago 8820808, Chile; satryl@veterinaria.uchile.cl; 3 Facultad de Medicina Veterinaria y Agronomía, Universidad de las Américas, Sede la Florida, Santiago 8242125, Chile; 4 Subdepartamento de Enfermedades Infecciosas, Instituto de Salud Pública de Chile, Santiago 7780050, Chile; jchormazabal@ispch.cl (J.C.H.); rhflores@ispch.cl (R.F.); 5 Unidad de Rehabilitación de Fauna Silvestre, Escuela de Medicina Veterinaria, Facultad de Ciencias de la Vida, Universidad Andrés Bello, Santiago 8370146, Chile; nicole.sallaberry@unab.cl

**Keywords:** *Brucella abortus*, *Brucella canis*, brucellosis, *Leptospira*, leptospirosis, wild canids, wildlife, *Lycalopex*

## Abstract

**Simple Summary:**

Wild canids play a crucial role in the environment, being an ecological agent of flora dispersal, top predators, and environmental sentinels for zoonotic emerging pathogens, such as *Brucella* spp. and pathogenic *Leptospira*. In Chile, three species of non-domestic native canids are present, and due to the growing concern about their interaction with humans and domestic animals, it is of utmost relevance to determine the role of these animals in the epidemiology of brucellosis and leptospirosis. This study aimed to detect the exposure to *B. abortus*, *B. canis*, and pathogenic *Leptospira* by serologic, bacteriologic, and molecular techniques in native foxes from rehabilitation and exhibition centers in Central Chile. Forty-six blood samples were obtained from culpeo and grey foxes, detecting exposure to *B. canis* and *L*. Javanica in 10.9% and 7.7%, respectively. Exposure was not registered by culture and qPCR in any of the sampled animals. Our results show for the first time in Chile exposure to *B. canis* in native foxes, highlighting the need to establish integrated surveillance programs to better evaluate the role of wild animals in the epidemiology of emerging zoonotic pathogens that may affect One Health.

**Abstract:**

*Brucella* *abortus*, *B. canis*, and pathogenic *Leptospira* are zoonotic pathogens that infect humans, as well as domestic and wild animals. In wild canids, they may affect their fertility and reproduction, threatening their conservation. Wild canids play a crucial role in the environment as meso- and top-predators and environmental sentinels for zoonotic pathogens. In Chile, three species of wild canids are present, and due to changes in land use and environmental dynamics, it is of utmost relevance to determine the role of these species in the epidemiology of brucellosis and leptospirosis. This study aimed to detect the exposure to *B. abortus*, *B. canis*, and pathogenic *Leptospira* by serologic, bacteriologic, and molecular techniques in native foxes from rehabilitation and exhibition centers in Central Chile. Forty-six blood samples were obtained from *Lycalopex culpaeus* and *L. griseus*, detecting 10.9% of seropositivity to *B. canis* and 7.7% to *L.* Javanica. No seropositivity was seen for *B. abortus*. Exposure was not registered by culture and qPCR in any of the sampled animals. Our findings are the first register of exposure to any *Brucella* species in wild canids in Chile and highlight the need to establish surveillance programs of these emerging pathogens.

## 1. Introduction

Land use influences disease (re-)emergence by changing the ecological dynamics of humans, wildlife, domestic animals, and pathogens [1,2]. These changes may favor encounters between populations that do not normally interact, allowing the spread of several pathogens that can seriously impact their conservation status. Moreover, some wild species may act as reservoirs of pathogens that threaten public and animal health, prohibiting the efficacy of control and eradication programs.

Among re-emerging zoonotic diseases, brucellosis and leptospirosis appear to be of high medical, veterinary, and economic impact [3,4]. In general terms, the epidemiology of these infections is well known, but the information about the role of wildlife on their dynamics is unclear, including primates, ungulates, and wild canids [4,5]. This lack of information contravenes the principles of One Health, where the animal-environment-human interface is crucial to maintain the health status worldwide [6].

In Chile, among their wildlife diversity, there are three species of native canids, including culpeo fox (*Lycalopex culpaeus*), grey fox (*L. griseus*), and Darwin’s fox (*L. fulvipes*). Still, only the first two inhabit the central zone of Chile [7]. Although *L. culpaeus* and *L. griseus* are under the category of conservation of least concern [8,9], the reduction and, in some cases, the destruction of their habitat due to conflict with livestock and forestry practices have led to a close interaction between foxes and free-ranging dogs. This interaction increases infectious agents’ transmission, which represents a potential risk of extinction [7,10,11].

Brucellosis is one of the most widespread zoonotic diseases worldwide, and is often neglected [12,13]. The genus *Brucella* comprises Gram-negative, aerobic, non-motile, non-spore-forming, and preferably intracellular coccobacilli. This genus is integrated by 11 species based on differences in their pathogenicity and host preferences [14,15], where *B. abortus*, *B. canis*, *B. melitensis*, and *B. suis* can infect canids and humans [16]. However, *B. canis* is recognized as the most frequently related to infection in canids [17]. Brucellosis caused by *B. abortus* is reported in virtually all countries where cattle are farmed. It constitutes a notifiable disease (ND) to the World Organization for Animal Health (OIE), for which reason many countries have implemented control and eradication programs in livestock [18]. Thus, some northern and central European countries, Australia, Canada, Japan, and New Zealand, are considered free [19], and in southern Europe prevalence is lower than 1% [20], while brucellosis remains as enzootic in Latin American countries, including Chile [21]. In the case of *B. canis*, as it is not considered within the ND, no official prevalence data are available, representing a challenge for studying its epidemiology. However, there is a worldwide presence in dogs, varying from 6% to 35% [22], but being considered exotic in Australia and New Zealand [23].

In Chile, native foxes could be naturally infected only by *B. abortus* and *B. canis*, given that the country has been free of *B. suis* and *B. melitensis* since 1987 and 2013, respectively [24]. *B. abortus* can be transmitted to wild canids by scavenging other infected animals, aborted fetuses, and placental membranes [25]. *B. canis* infection occurs between dogs by venereal transmission, but also by the conjunctival and oronasal routes, through contact with abortion products, vaginal secretions, milk, seminal fluids, and urine [26].

Despite the fact that brucellosis has been poorly studied in domestic and wild canids, *B. abortus* and *B. canis* can produce reproductive clinical signs, including late abortion, birth of weak litters, epididymitis, orchitis, and prostatitis [27]. Nevertheless, it is not clear whether wild canids are enhancers or carriers [15].

Similarly, leptospirosis is a widespread global zoonotic disease caused by several species of the *Leptospira* genus, including more than 260 serovars [28]. This genus comprises Gram-negative, motile, obligate aerobes spirochaetes. These bacteria are capable of causing systemic disease in a wide variety of hosts, including humans, and wild and domestic animals, characterized by fever, renal and hepatic insufficiency, pulmonary manifestations, and reproductive failure [28,29]. Leptospirosis in humans and other animals is transmitted by exposure to water or soil contaminated by urine of infected animals, or by directly contacting it [30]. Additionally, several species, including dogs, are considered carriers of pathogenic *Leptospira* strains [28].

Several studies have reported exposure to pathogenic *Leptospira,* ranging from 9.9% to 47% in red foxes (*Vulpes vulpes, V. fulva*) [29,31,32], 31% in grey foxes (*Urocyon cinereoargenteus*) [31], and 8.4% in wolves (*Canis lupus*) [32]. In Chile, only two studies have addressed the exposure to different *Leptospira* serovars in native canids. Thus, in native foxes from Tierra del Fuego, Southern Chile, Moya et al. [33] registered 20% in *L. culpaeus lycoides*, including serovars Ballum, Australis, Autumnalis, Borincana, and Icterohaemorrhagiae; and 8% to *L*. Autumnalis in *L. griseus*. In the second, Llanos-Soto et al. [34] reported the detection of *L*. Ballum and *L*. Canicola in a *L. culpaeus* from Central Chile.

On the other hand, exposure to *Brucella* spp. in wild canids is about 40% in coyotes (*C. latrans*) [35], 42% in wolves [36], 43% in black-backed jackals (*C. mesomelas*) [37], and 40% in red foxes [38]. In Chile, to date, there are only two serological studies in native foxes. Olivares et al. [39] obtained samples from 158 animals from the Metropolitan Zoo of Santiago, where no native canids were seropositive. In the second study, Moya et al. [33] analyzed 27 sera obtained from foxes from Tierra del Fuego to detect anti-*B. canis* antibodies, with no seropositive animals. Overall, in most of these studies different serological tools were used, which may lead to underestimation of the circulation of these pathogens and prevent comparison of their prevalence. 

Considering the more frequent interaction between native canids and domestic and stray dogs, particularly during COVID-19 lockdowns, it is important to determine the presence of these pathogens in Chilean native foxes. Conservation of native canids is of utmost relevance considering that these species play a crucial role in the environment, being ecological agents of flora dispersal and meso- and top-predators. Moreover, their ecological role indirectly increases the diversity of ecosystems and contributes to the prevention of spreading infectious diseases. Thus, this study aimed to detect the exposure to pathogenic *Leptospira*, *B. abortus*, and *B. canis* in native foxes of Central Chile by bacteriological, serological, and molecular techniques, to elucidate its role on the epidemiology of leptospirosis and brucellosis and their ecological impact, under the concept of One Health.

## 2. Materials and Methods

### 2.1. Sample Collection

Sampling was performed with prior institutional (permit code 19259-VET-UCH) and signed consent in two zoos and four wildlife rehabilitation centers in Central Chile during 2019–2020. Samples were obtained through cephalic or jugular venipuncture from native foxes over one year of age, without antimicrobial therapy during the previous four weeks, and not pregnant or in the process of lactation. At least 3 mL of whole blood was collected by veterinarians in Vacutainer^®^ Heparin Blood Collection Tubes (Becton, Dickinson & Co., Franklin Lakes, NJ, USA) and at least 1 mL of blood in Vacutainer^®^ Serum Blood Collection Tubes (Becton, Dickinson & Co., Franklin Lakes, NJ, USA). All foxes were clinically examined at the time of blood collection, primarily for signs suggestive of reproductive disease. Among these signs, we looked for vaginal discharge, orchitis, epididymitis, scrotal dermatitis, testicular atrophy, as well as abortion, reproductive failure, stillbirth, and infertility records in zoo animals. After collection, all samples were immediately refrigerated and transported to the laboratory within four h.

### 2.2. Detection of Antibodies against B. abortus, B. canis, and Pathogenic Leptospira

All blood samples collected in serum tubes were centrifuged at 5000× *g* (Labofuge 200, Marshall Scientific, Hampton, NH, USA) for 10 min to separate serum from clotted blood. Next, the presence of antibodies against *B. canis* was determined by counterimmunoelectrophoresis (CIEF), using LPS-R of *B. ovis* as antigen, at 200 V and 30 mA for 90 min [40]. Serum previously obtained from a bitch experimentally inoculated with the *B. canis* str. RM 666 was used as positive control [41]. The presence of antibodies against *B. abortus* was detected by the Rose Bengal test (RB) according to the Technical Instructions for the Analysis of Rose Bengal of the Servicio Agrícola y Ganadero de Chile (SAG) [42]. A bovine serum positive for RB from our collection was used as positive control.

Detection of antibodies against pathogenic *Leptospira* was carried out in the Instituto de Salud Pública de Chile (ISP) by the microscopic agglutination test (MAT). In this MAT sera were confronted with a panel of 19 serovars of *Leptospira* strains in Ellinghausen-McCullough-Johnson-Harris medium. Table 1 shows the *Leptospira* serovars used in the MAT. Thus, serum samples were diluted 1:50, and 50 μL of this diluted serum and 50 μL of diluted antigen were added to a flexible plastic U-bottom microplate and incubated at 28 °C ± 1 °C for two h. The reading was carried out in a dark field microscope with a 10× objective. Samples were considered reactive when agglutination of at least 50% of leptospires was observed [33]. Reactive samples were serially diluted to determine titer, starting from 1:50 to 1:3200.

### 2.3. Bacteriological Detection of B. abortus and B. canis

All blood samples collected in tubes with anticoagulant were analyzed in a class 2A biosafety cabinet (Heal Force Safe 1200, Shanghai, China) with prior institutional biosecurity permission (permit code 114-VET-UCH) by microbiological culturing, according to Alton et al. [43] and Keid et al. [44] for the detection of *B. abortus* and *B. canis,* respectively. Briefly, 3 mL of blood were added into 30 mL of 3% trypticase soy broth (Becton, Dickinson & Co., Franklin Lakes, NJ, USA) with sodium citrate (Merck^®^, Darmstadt, Germany) to a final concentration of 2% and pH 7.4. Twelve mL of this broth were incubated at 37 °C for 30 days with 5% CO_2_ for the culture of *B. abortus*, and the remaining volume was incubated at 37 °C for 30 days in aerobiosis for the culture of *B. canis*. Every seven days, 100 μL of those incubated broths were plated with Digralsky loop (Biologix^®^, Lenexa, KS, USA) onto *Brucella* agar (Becton, Dickinson & Co., Franklin Lakes, NJ, USA) plates supplemented with cycloheximide (100 mg/L, Merck^®^, Darmstadt, Germany), bacitracin (25,000 IU, Merck^®^, Darmstadt, Germany), and polymyxin B (6000 IU, Merck^®^, Darmstadt, Germany). Then, each plate was incubated at 37 °C for at least 72 h, using the same conditions as explained above. *B. canis* SCL strain [45] and the field *B. abortus* isolate kindly donated by the SAG were used as quality controls of the *Brucella* agar plates. The method used at our lab for *Brucella* spp. detection in culture is as follows: primary screening of colonies through Gram staining and agglutination with monospecific sera anti-A (*B. abortus* str. 1119-3) and anti-R (*B. canis* str. RM 666) [46]. If positive colonies are present, then those colonies are cultured individually onto *Brucella* agar plates (Becton, Dickinson & Co., Franklin Lakes, NJ, USA), as described above, for further identification [46]. A further qPCR analysis is always performed in the colonies. Positive and negative controls are used throughout the experiment. For the positive controls, we used Chilean *B. abortus* and *B. canis* isolates, as noted above.

### 2.4. Molecular Identification of B. abortus and B. canis Colonies

All *B. abortus* and *B. canis* colonies were subjected to real-time PCR (qPCR) for further confirmation. Thus, five colonies per plate were suspended in 500 μL of sterile nuclease-free water and boiled for 15 min at 100 °C. A total volume of 200 μL of lysed bacteria per sample was used to purify DNA using the NucleoSpin^®^ Plant II kit (Macherey-Nagel^®^, Düren, Germany) following the manufacturer’s instructions. The concentration and quality of extracted DNA were determined by a Nanodrop spectrophotometer with EPOCH equipment (BioTek, Winooski, VT, USA) and stored at −20 °C until further use.

The purified DNA was used to perform qPCR analysis using primers and reaction conditions previously described for *B. canis* [47], while primers for *B. abortus* were designed with Primer3 software v 0.4.0 (http://frodo.wi.mit.edu/primer3/) using available *B. abortus* chromosome I genome sequence (AE017223.1) (Table 2). The standard curves of *B. abortus* primers were evaluated using serial dilutions of the DNA of the field isolate of *B. abortus* donated by the SAG, with amplification until at least 1:1000 of the bacterial DNA. Additionally, a melt curve analysis was performed to determine specific amplifications of *B. abortus* primers. The qPCRs were performed using a final volume of 10 μL with five μL of KAPA SYBR FAST qPCR Master Mix (Kapa Biosystems Inc., Wilmington, MA, USA), 0.5 M of forward primer, 0.5 M of reverse primer, and 4 μL of sample DNA. qPCR reactions were performed on a Rotor-Gene Q real-time PCR cycler (Qiagen, Hilden, Germany), and the results analyzed by the Rotor-Gene Q series software (Qiagen, Hilden, Germany), determining the cycle threshold (Ct) values. The samples were considered as positive for the presence of *B. abortus* and *B. canis* if they had a Ct value lower than 30 cycles, as empirically obtained. A standard curve was generated using a 10-fold dilution series of DNA with six points, in triplicate, using nuclease-free water as a negative control; the blood DNA of a non-infected dog previously obtained [48] was used as an internal negative control, and DNA from *B. canis* SCL strain [45] and a field isolate of *B. abortus* kindly donated by the SAG were used as positive controls. All the qPCR assays were carried out complying with the Minimum Information for the Publication of Quantitative Real-Time PCR Experiments guidelines [49].

### 2.5. Molecular Identification of B. abortus, B. canis, and Pathogenic Leptospira from Blood

All samples collected in Vacutainer^®^ Heparin Blood Collection Tubes (Becton, Dickinson & Co., Franklin Lakes, NJ, USA) were processed as described previously [48] for further analysis. Briefly, blood DNA extraction was performed using the NucleoSpin^®^ Plant II kit (Macherey-Nagel^®^, Düren, Germany), and concentration and quality of extracted DNA was determined by a Nanodrop spectrophotometer with EPOCH equipment (BioTek, Winooski, VT, USA) and stored at −20 °C. This protocol of DNA extraction has successfully avoided possible effects of heparin on bacterial DNA recovery or qPCR amplification in dog’s blood [48]. The DNA extracted from blood was used to perform the qPCR to identify *B. abortus*, *B. canis*, and pathogenic *Leptospira.* For the detection of *B. abortus* and *B. canis*, the qPCR conditions for blood samples analysis were the same previously described (Table 2). The qPCR analysis for detecting pathogenic *Leptospira* used primers and reaction conditions previously described by Bourhy et al. [50], using DNA from field isolates of *L.* Pomona and *L*. Canicola kindly donated by the SAG as positive controls.

## 3. Results

During the sampling period, 46 samples were obtained. Eight (17.4%) corresponded to exhibition centers and 38 (82.6%) to wildlife rehabilitation centers. Sampled animals included 33 (71.7%) *L. culpaeus* and 13 (28.3%) *L. griseus*. The animal’s age could be determined in 89.1% (*n* = 41) of the individuals, mostly adults (58.5%, *n* = 24). On the other hand, sex could be registered in 67.4% (*n* = 31) of the animals, with 54.8% (*n* = 17) males and 45.2% (*n* = 14) females. Additionally, no animals showed any suggestive signs of brucellosis or leptospirosis at the sampling time.

From the 46 animals analyzed, antibodies against *B. canis* were detected in five *L. culpaeus* (10.9%) individuals, primarily females from wildlife rehabilitation centers. No anti-*B. abortus* antibodies were detected in any of the samples. Only 13 samples could be subjected to MAT due to the scarce volume of sera obtained, while 12 turned negative (titer < 1:50). A single juvenile female *L. culpaeus* from a wildlife rehabilitation center was positive to anti-*L*. Javanica antibodies (titer 1:100), but not seropositive to *B. canis* antibodies. On the other hand, all blood samples were negative to *B. abortus* or *B. canis* bacterial culture. Table 3 shows the epidemiological characteristics of the *B. canis* seropositive animals.

Regarding the molecular detection of the assessed pathogens directly on blood, no amplification in any of the samples was obtained in the qPCRs (Ct > 35). However, all positive controls and their dilutions amplified, with Ct values ranging between cycle 9 and 27. Additionally, the standard curves for each set of primers were in optimal range, with an efficiency >98%, and R2 over 0.999, 0.997, and 0.999 for *B. canis*, *B. abortus*, and pathogenic *Leptospira*, respectively.

## 4. Discussion

The spread and persistence of newly emerged (or re-emerged) pathogens can be perpetuated by a combination of factors, including expanding global human populations and urbanization, international trade and travel, intensive livestock husbandry systems, proliferation of reservoir populations, and antimicrobial drug use, among others [51,52].

Brucellosis and leptospirosis are important worldwide-spread infectious diseases of animal origin. These diseases affect the economy and public health, and several wild species act as reservoirs [3,4]. Therefore, their control and eradication depend on efficient and rapid detection and surveillance, focusing not only on their classical domestic hosts but addressing complex multi-host systems, including wild animal species [4,53]. In this sense, wild carnivores, primarily predators and/or scavengers, interact with a wide range of wild and domestic species, thus representing good sentinels of several pathogens in their natural environment [54].

Here, we detected a 10.9% of seropositivity against *B. canis* employing CIEF. Results were higher than expected and represent the first detection of seropositivity to this pathogen in native foxes in our country, suggesting that Chilean native foxes are exposed to *B. canis* in their natural environments. Specifically, the CIEF technique detects antibodies until day 266 post-infection in experimentally infected dogs [41], and possesses a sensitivity and specificity of 100% and 96.8%, respectively, when compared to agar gel immunodiffusion [55,56], and of 80% and 94.6%, respectively, when compared to bacteriological culture [57]. Additionally, it should be considered that in dogs antibody titers vary according to the phase of the infection, decreasing considerably in chronically infected animals, thus being scarcely detected by these methods [58]. The method of choice to isolate *B. canis* from infected animals is blood culture; however, the animal must be bacteremic to obtain a positive result [48]. Therefore, a single negative blood culture does not rule out a possible infection [44], and performing serial cultures is required [16]. This fact could explain our positive results at serology, but negative to bacterial culture, even when samples were incubated for 42 days. Moreover, considering that, to date, this pathogen has not been isolated from wild canids [59,60], Moreno [15] proposed that *B. canis* evolved in the dog’s ancestor after its predation on *B. suis* biovar 4-infected animals, which may explain the negative results of blood cultures. This supports the idea that these animals act as terminal hosts rather than reservoirs.

In the case of *B. abortus*, positive cases from detecting antibodies against this pathogen have been reported in Latin American countries from dogs [61,62] and foxes [63]. However, in gray foxes (*U. cinereoargenteus*) experimentally infected with *B. abortus*, seroconversion occurs around 14 days after exposure and declines between 35–49 days post-infection [64]. Therefore, if foxes analyzed here could have been recently or chronically infected, seropositivity could be hardly detected. It is important to note that RB is a simple and accurate serological test recommended by the OIE. Still, the lack of validated tests for wildlife species remains a major limitation for these studies [65]. Another factor that may explain our results is that in Chile, since 1991, there has been an official bovine brucellosis eradication program and constant epidemiological surveillance. Thus, in 2010 the SAG reported a prevalence of *B. abortus* infection of 1.3 per 10,000 animals [24]. This low prevalence of bovine brucellosis could explain the null detection of this pathogen in the present study. Despite this, the official surveillance is only carried out in cattle, which could cause a re-emergence of the disease if exhaustive surveillance is not carried out in possible wild reservoirs, which could potentially transmit *B. abortus* to cattle. This situation was early reported by Davis et al. [66], where coyotes were experimentally fed with bovine fetal and placental tissues inoculated with *B. abortus*, and seropositive-confirmed individuals were placed together with six seronegative parturient heifers. After 14 days of being placed together with the infected coyotes three heifers turned seropositive, and since 35 to 65 days post-exposure all of these animals aborted. Additionally, *B. abortus* was isolated from vaginal swabs, milk, and placental and fetal tissues of the seropositive heifers; and from the spleen, retropharyngeal lymph nodes, superficial inguinal lymph nodes, and palatine tonsils from the coyotes *post-mortem*.

Conversely, here we did not isolate *B. abortus* in any of the sampled animals. This could be due to the absence of bacteremia, among other issues [43]. Indeed, Tessaro & Forbes [67] reported the isolation of this pathogen from wolves (*C. lupus*) by blood culture. In this study, the authors fed four wolves with canned dog food experimentally contaminated with *B. abortus* biovar 1, and registered the presence of the pathogen in all blood clots cultures, with bacterial counts ranging from 2 to 8 CFU/g, until day 21 post-infection. On the other hand, only one animal tested positive to urine culture, at 12 h post-infection. These results support the use of blood cultures to detect *B. abortus* in wild canids, considering that isolation rates could be improved by using highly selective media and prolonged incubation periods [43], like those included in our study.

Some serovars of *Leptospira* can chronically infect domestic and wild animals, including wild canids [68,69]. Particularly in South America, infection with this pathogen has been registered in wild canids in Argentina [69] and Central Chile [34], where serovars Ballum, Canicola, Icterohaemorragiae, Castellonis, and Grippotyphosa were detected. For the first time, we reported the presence of antibodies against *L*. Javanica in *L. culpaeus*. This serovar has been detected mainly in rodents [70,71], but also in dogs [72], horses [73], and cattle [74], especially in tropical countries. Seropositivity could be due to the presence of this pathogen in the environment of these foxes (e.g., water sources), in some reservoir animals that constitute their diet (e.g., rodents), or by contact with infected domestic animals. Our detection rate of anti-*Leptospira* antibodies could have been related to the infection phase of the sampled animals. In this context, Reilly [75] intraperitoneally infected seven red foxes (*V. vulpes*) with *L*. Grippotyposa, detecting antibodies from day 21 until day 42 post-infection. Thus, animals infected with pathogenic *Leptospira* could be seronegative by traditional techniques. Although the detection of leptospiral antibodies through MAT remains the gold standard for leptospirosis diagnosis [76], it may not assess all the Leptospira serovars circulating in specific areas. Therefore, other undetected serovars may be present in the sampled animals, a question that must be further investigated. The panel assessed here included the most detected *Leptospira* serovars in dogs in Chile, namely Automnalis, Ballum, Canicola, Hardjo, Icterohaemorragiae, and Pomona [77,78,79].

Despite registering seropositivity for *B. canis*, we did not detect any positive animals based on the qPCR analysis. Previously, we reported a 100% of consistency between the qPCR used here to detect *B. canis* directly on blood from dogs positive to bacterial isolation, with no amplification inhibition. At the same time, no amplification was detected in seropositive but bacteriologically negative dogs [48]. Our results could be explained by the absence of the pathogen in blood due to intermittent bacteremia or to the specific co-evolution of *B. canis* and domestic dogs [15,60].

A single *L. culpaeus* was positive to *L*. Javanica but negative to its detection by qPCR. It has been reported that in experimentally infected red foxes, the leptospiremic phase lasts about a week [75]. In this period, leptospires can be isolated from blood and tissues. It must be mentioned that the qPCR protocol’s reported sensitivity is 10^3^ bacteria/mL with a 100% of specificity detecting pathogenic *Leptospira* on blood [50]. Overall, these results suggest that the seropositive animals were in a chronic phase or just exposed to these pathogens. To further elucidate this, serial sampling should be considered.

## 5. Conclusions

These findings constitute the first study to register exposure to *B. canis* in native canids in Chile, providing relevant information for studying the epidemiology of these diseases in wild animals. Moreover, considering the small sample size used, we detected a number of seropositive *L. culpaeus*. Therefore, it is likely that this pathogen is widely present in their natural environments. Our results highlight the role of Chilean native foxes as environmental sentinels for zoonotic emerging pathogens, but further studies are needed to describe this situation in free-ranging canids. In order to assess the distribution of these pathogens and their impact in the conservation of wild populations, serial sampling is recommended. Therefore, it is essential to establish integrated surveillance programs, both national and regional, ensuring a One Health approach.

## Figures and Tables

**Table 1:** List of the 19 *Leptospira* serovars used in the MAT to detect anti-*Leptospira* antibodies in sera obtained from native foxes.

Serovar	Species	Strain
Andamana	*L. biflexa*	CH11
Australis	*L. interrogans*	Ballico
Autumnalis	*L. interrogans*	Akiyami A
Ballum	*L. borgepetersenii*	Mus 125
Bataviae	*L. interrogans*	Van Tienen
Canicola	*L. interrogans*	Hond Utrecht IV
Celledoni	*L. weilii*	Celledoni
Cynopteri	*L. kischneri*	3522 C
Djasiman	*L. interrogans*	Djasiman
Grippotyphosa	*L. interrogans*	Moskva V
Borincana	*L. santarosai*	HS 622
Icterohaemorragiae	*L. interrogans*	RGA
Javanica	*L. borgepetersenii*	Veldrat Batavia 46
Georgia	*L. santarosai*	LT117
Pomona	*L. interrogans*	Pomona
Pyriogenes	*L. interrogans*	Salinem
Canicola	*L. interrogans*	Reubush
Copenhageni	*L. interrogans*	M20
Hardjo	*L. interrogans*	Hardjoprajitno

**Table 2:** Primer sequences, Tm and source for qPCR analysis of *B. abortus*, *B. canis*, and pathogenic *Leptospira*.

Species	Forward	Reverse	Tm	Source
*B. canis*	ACGAACACAAGGGCCAATAC	GGACGGCTACAAGATCGAAG	62	[47]
*B. abortus*	CGCTCGCTGCTAAAGACATA	TAGGATCGACCTCGACAATACA	62	This study
Pathogenic *Leptospira*	AAGCATTACCGCTTGTGGTG	GAACTCCCATTTCAGCGATT	62	[50]

**Table 3:** Epidemiologic characteristics of the *B. canis* seropositive native foxes.

Animal ID	Animal Species	Center of Origin	Age Class	Sex
LC-10	*L. culpaeus* *	Exhibition 1	Adult	Female
LC-18	*L. culpaeus*	Rehabilitation 3	Juvenile	Female
LC-21	*L. culpaeus*	Rehabilitation 2	Juvenile	Female
LC-23	*L. culpaeus*	Rehabilitation 1	Adult	Male
LC-49	*L. culpaeus*	Rehabilitation 1	Adult	Female

* The origin of this individual was a wildlife rehabilitation center, but kept afterward in exhibition.
